# Case Report: “*DEK::AFF2* fusion associated sinonasal carcinomas: a novel oncogenic driver and emerging therapeutic strategies”

**DOI:** 10.3389/fimmu.2025.1611790

**Published:** 2025-07-04

**Authors:** Moritz Knebel, Abbas Agaimy, Jan Philipp Kühn, Sandrina Körner, Felix Braun, Lukas Brust, Veronika Flockerzi, Silke Wemmert, Benedikt Balensiefer, Bernhard Schick, Umut Yilmaz, Malek Zaito, Alessandro Bozzato, Maximilian Linxweiler

**Affiliations:** ^1^ Institute of Otorhinolaryngology, Saarland University, Homburg, Germany; ^2^ Department of Otorhinolaryngology, Head and Neck Surgery, Saarland University Medical Center (UKS), Homburg, Germany; ^3^ Institute of Pathology, Erlangen University Hospital, Friedrich Alexander University of Erlangen-Nuremberg, Comprehensive Cancer Center Erlangen-Europäische Metropolregion Nürnberg (CCC ER-EMN), Erlangen, Germany; ^4^ Departement of Oncology, Hematology, Clinical Immunology, and Rheumatology, Saarland University Medical Center (UKS), Homburg, Germany; ^5^ Department of Diagnostic and Interventional Neuroradiology, Saarland University Medical Center (UKS), Homburg, Germany; ^6^ Departement of Nuclear Medicine, Saarland University Medical Center (UKS), Homburg, Germany

**Keywords:** *DEK::AFF2* fusion, sinonasal cancer, neoadjuvant chemo(radio)therapy, prognosis, diagnostic

## Abstract

**Background:**

*DEK::AFF2* fusion-associated carcinomas of the sinonasal tract are exceedingly rare, with fewer than 100 cases reported worldwide, but probably underrecognized. Recently classified by the WHO as a distinct provisional subtype of non-keratinizing squamous cell carcinoma, these tumors pose significant diagnostic and therapeutic challenges. Their histological resemblance to inverted papillomas and their bland histology in most cases often leads to misdiagnosis, while their aggressive behavior underscores the need for a tailored treatment approach.

**Case presentation:**

We report two cases of *DEK::AFF2* fusion-associated carcinomas managed at Saarland University Medical Center. The first case involved a 46-year-old woman who initially presented with recurrent sinonasal inverted papilloma, confirmed through multiple surgical interventions over nearly a decade. In 2023, reevaluation and genetic analysis revealed a *DEK::AFF2* fusion. The patient demonstrated an exceptional response to three cycles of neoadjuvant gemcitabine and cisplatin, achieving complete remission on MRI restaging. This allowed a shift to definitive chemoradiotherapy, with sustained disease-free status confirmed by a PET-CT three months post-treatment in July 2024. The second case involved a 66-year-old woman presenting with recurrent inverted papilloma affecting the sinonasal and tympanic regions. Despite multiple surgeries, malignant transformation to invasive squamous cell carcinoma occurred, with lymph node metastasis and intracranial spread. A combined otolaryngological and neurosurgical approach was undertaken, but the disease progressed. The patient passed away in January 2020, with postmortem review of the prior histology and genetic analysis confirming *DEK::AFF2* fusion carcinoma that showed bland-looking papilloma-like morphology in the initial specimens and later a high-grade cytology indicating biological progression to poorly differentiated carcinoma.

**Conclusion:**

These cases highlight the aggressive nature of *DEK::AFF2* fusion-associated carcinomas and the critical role of genetic profiling in diagnosis and management. The exceptional, first ever reported response to neoadjuvant chemotherapy in one case underscores the potential for personalized treatment strategies, warranting further investigation into targeted therapies for this rare malignancy.

## Introduction

The adage, “When you hear hoofbeats, think of horses, not zebras,” attributed to Theodore Woodward, emphasizes the importance of considering common diagnoses first. While this principle applies to most clinical scenarios, the following cases underscore the need to occasionally consider zebras too. The detection of a *DEK::AFF2* gene fusion in sinonasal neoplasms remains exceedingly rare, with fewer than 100 reported cases globally (1, 2). This novel entity, now classified by the WHO as a distinct, provisional subtype of non-keratinizing squamous cell carcinoma (SCC) of the sinonasal tract, is histologically characterized by syncytial nests of monomorphic cells, papillary growth patterns, inverted ribbons, and vague peripheral palisading, closely mimicking exophytic and endophytic (inverted) papillomas (3). *DEK::AFF2* fusion-associated carcinomas are often initially misdiagnosed as inverted sinonasal papillomas due to their deceptively benign histological appearance (4). However, they exhibit aggressive behavior, with frequent local recurrences, cervical lymph node metastases, and distant metastases, highlighting their malignant potential (5, 6). Notably, as previously described in a single case report, *DEK::AFF2* carcinomas demonstrate an exceptional response to anti-PD-1 immunotherapy, despite negative PD-L1 staining and a low tumor mutational burden (7). The particular molecular signature of *DEK::AFF2* sinonasal fusion-associated carcinomas come along with unique clinical challenges, especially due to the absence of standardized therapeutic protocols. This case report, detailing two patients treated at the Saarland University Medical Center, aims to expand current knowledge on the diagnostic approaches and therapeutic strategies available for managing this rare malignancy. By elucidating these cases, we seek to contribute to the evolving understanding of this tumor entity and its potential treatment pathways.

## Materials and methods

### Targeted RNA sequencing for the detection of the *DEK::AFF2* fusion

**Figure 1:**
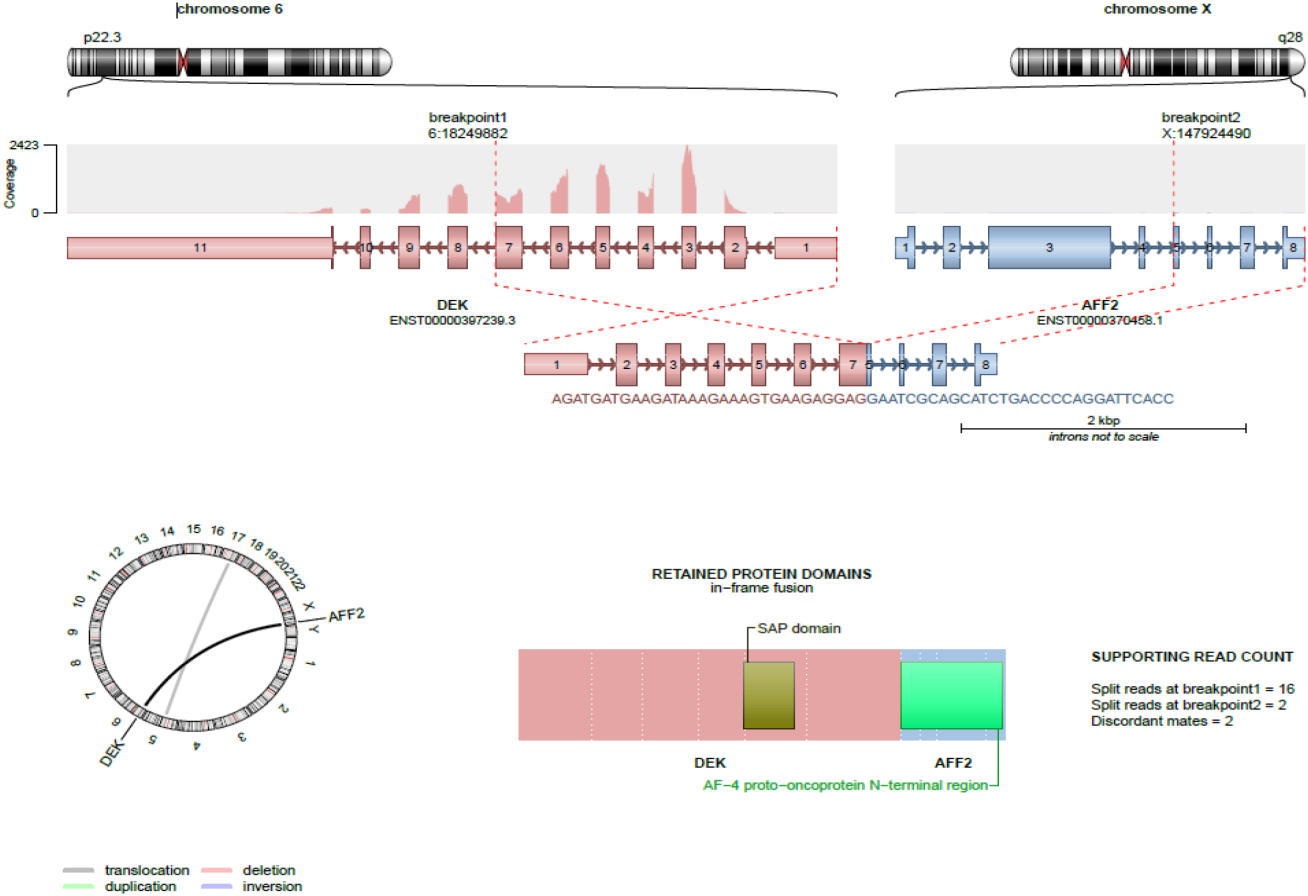
Visualization of the *DEK::AFF2* fusion from RNA sequencing using Arriba. Structural and functional characteristics of the fusion are shown. Structure of the fusion transcripts, protein domains retained in the fusion protein and topology are visualized.

The tissue specimens were fixed in formalin and processed routinely for histopathology. For *DEK::AFF2* fusion detection, RNA was isolated from formalin-fixed paraffin embedded (FFPE) tissue sections using RNeasy FFPE Kit of Qiagen (Hilden, Germany) and quantified spectrophotometrically using NanoDrop-1000 (Waltham, United States). Molecular analysis was performed using the TruSight RNA Fusion panel (Illumina, Inc., San Diego, CA, USA) with 500 ng RNA as input according to the manufacturer`s protocol. Libraries were sequenced on a MiSeq (Illumina, Inc., San Diego, CA, USA) with > 3 million reads per case, and sequences were analyzed using the RNA-Seq Alignment workflow, version 2.0.1 (Illumina, Inc., San Diego, CA, USA). The Illumina and Arriba softwares were used for the detection and visualization of fusions from RNA sequencing, as shown in **Figure 1** respectively (8).

## Case presentation

### Case 1

In 2014, a 46-year-old woman with a history of vulvar cancer but otherwise without significant comorbidities underwent cranial magnetic resonance imaging (MRI), which incidentally revealed findings consistent with chronic rhinosinusitis involving the sphenoid and ethmoidal sinuses, along with suspected erosion of the frontal skull base. Surgical intervention was subsequently performed at our department with the histopathological finding of an inverted papilloma.

**Figure 2:**
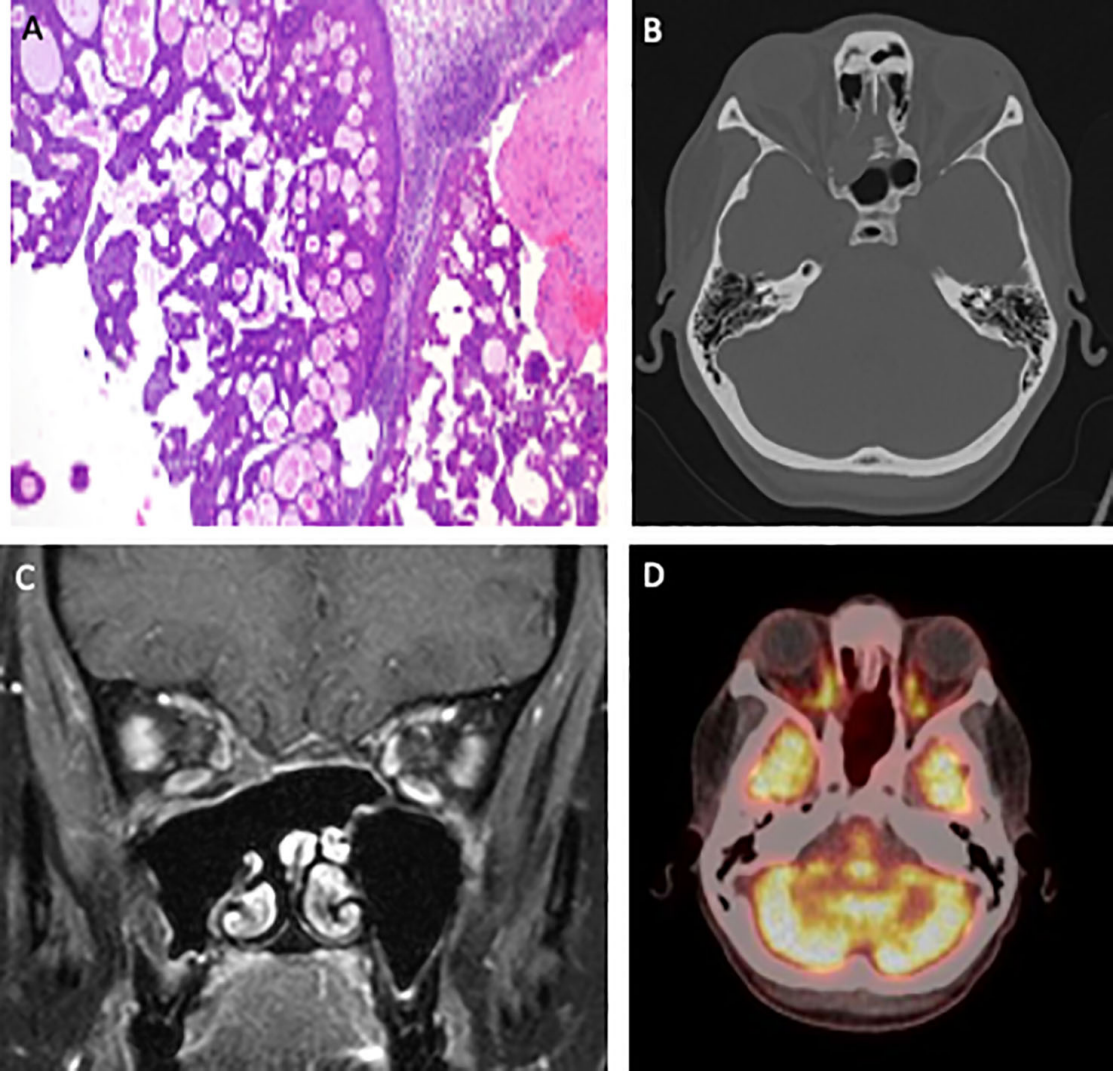
Visualization of the therapeutic trajectory, including a representative histological image (magnification ×100) illustrating a combined exophytic-endophytic papilloma-like growth with bland histopathology **(A)**, as well as morphological imaging depicted through a pretherapeutic CT scan obtained in June 2023 **(B)**, an MRI scan in February 2024 showing an excellent response to induction chemotherapy **(C)**, and a post-therapeutic PET-CT scan demonstrating a complete response in July 2024 **(D)**.

Following the initial surgery, the patient experienced recurrent symptoms and radiological findings indicative of disease persistence, necessitating additional interventions. Between 2014 and 2016, three further functional endoscopic sinus surgeries (FESS) were performed including drilling at the pathology base at our department due to morphological evidence of recurrent papilloma involving the sphenoid and ethmoidal sinuses, with progressive exposure of the dura. Histopathological evaluations of these specimens consistently confirmed the diagnosis of inverted papilloma (see **Figure 2A**).

In early 2017, MRI findings again raised suspicion for disease recurrence. The patient underwent another FESS procedure at an external institution, with histopathological analysis once more confirming recurrent inverted papilloma. Further disease progression led to an additional surgery at our department in 2018, followed by two more FESS procedures performed at another institution through 2019.

In 2023, surveillance computed tomography (CT) revealed new findings of recurrent inverted papilloma with erosion of the lamina papyracea and extension into the orbital cavity (**Figure 2B**). This aggressive behavior prompted comprehensive genetic analysis following the most recent surgical intervention in June 2023. The analysis identified a *DEK::AFF2* fusion, a rare genetic alteration potentially underlying the patient’s refractory disease course.

As no lymph node involvement or distant metastases were detected, the initial treatment strategy proposed by the interdisciplinary tumor board focused on neoadjuvant therapy. The patient was scheduled to receive three cycles of gemcitabine and cisplatin, with the aim of achieving an R0 resection via a combined neurosurgical and ENT surgical approach.

In February 2024, however, MRI restaging revealed a complete response to induction chemotherapy (see **Figure 2C**). Given this first ever reported, exceptional response, the treatment approach was adapted to definitive primary chemoradiotherapy (CRT). This consisted of five cycles of cisplatin alongside targeted radiation therapy encompassing the nasal sinuses, partial orbital involvement, and the lymphatic drainage regions of levels II and III bilaterally.

Follow-up imaging in July 2024, using PET-CT (see **Figure 2D**), demonstrated complete metabolic remission of the *DEK::AFF2* fusion-associated sinonasal carcinoma. Since then, the patient has been enrolled in regular oncological surveillance with no evidence of disease recurrence to date.

### Case 2

**Figure 3:**
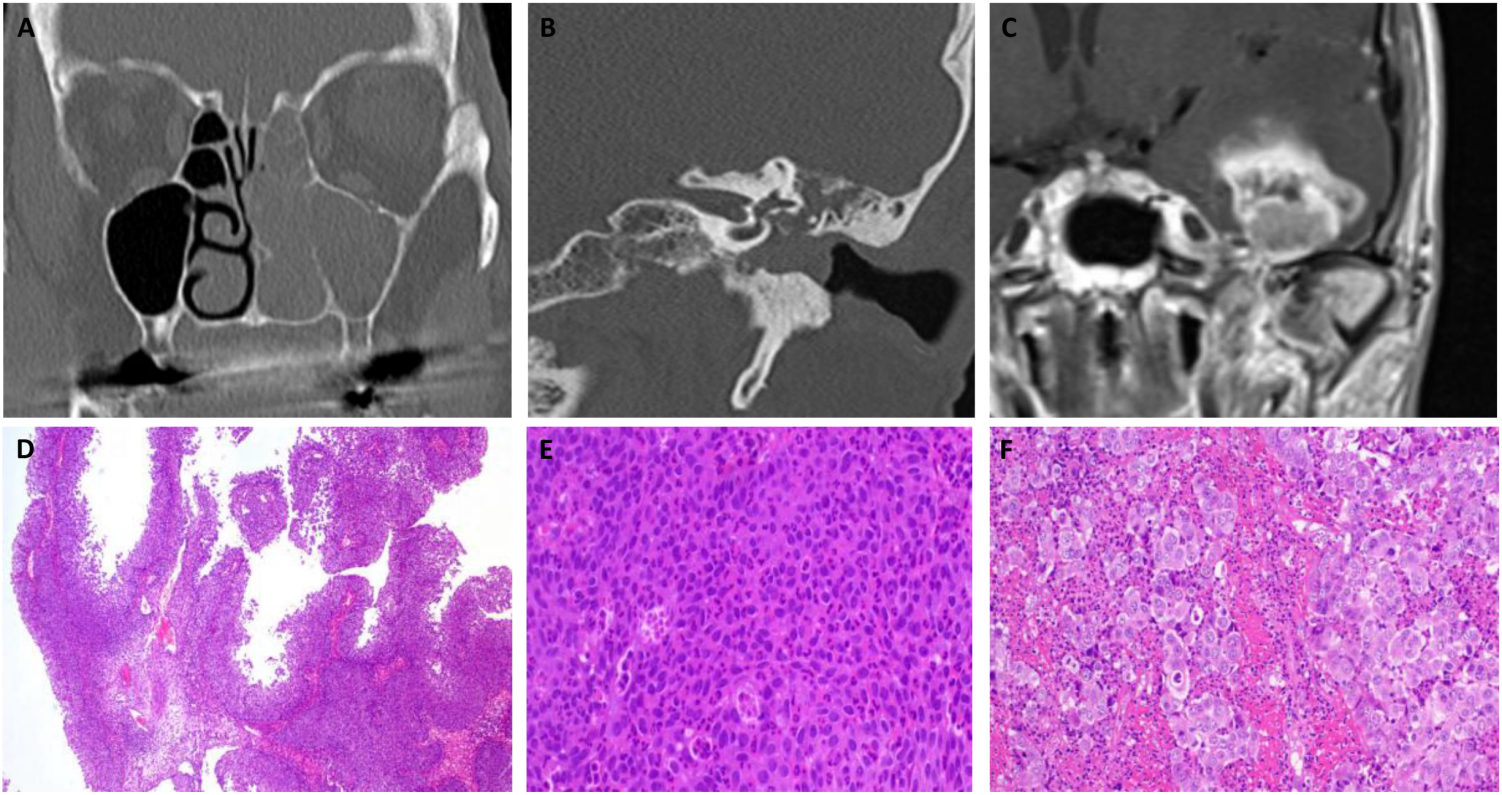
Sequential morphological imaging illustrating disease progression in Case 2, beginning with a CT scan in 2016 identifying an indeterminate mass in the nasal cavity and sinuses **(A)**, followed by a CT scan in July 2017 revealing radiologic features consistent with squamous cell carcinoma in the left tympanic cavity **(B)**, and an MRI in 2019 confirming metastatic dissemination to the temporal lobe **(C)**. Representative histological images of Case 2, illustrating the initial papillary growth with bland cytomorphology (magnification: x100) **(D)**, notable excessive neutrophilic infiltration within the tumor (magnification: x400) **(E)**, and transformation in later recurrences to a high-grade large-cell squamous cell carcinoma, which ultimately led to the patient’s death (magnification: x400) **(F)**.

The second case involved a 66-year-old woman who initially presented with recurrent episodes of bloody rhinorrhea and an unclear mass in the nasal cavity. The patient was otherwise healthy, with no known history of neoplastic disease. CT-imaging revealed involvement of the left maxillary, ethmoidal, and frontal sinuses (see **Figure 3A**). Histopathological examination after performed FESS confirmed the diagnosis of an inverted papilloma. In May 2017, six months after the initial presentation, the patient returned with progressive conductive hearing loss. An unclear mass involving the tympanic cavity, left maxillary sinus, and the posterior nasal septum was shown in CT-imaging (see **Figure 3B**). A tympanoplasty with endaural tumor excision and revision FESS was performed. Histopathological examination identified an inverted papilloma affecting both the sinonasal and tympanic regions.

Due to the incomplete excision of the tympanic lesion, a combined endaural and retroauricular approach was employed to achieve tumor debulking. Despite this intervention, routine otoscopic follow-up in August 2017 identified a suspected recurrence of inverted papilloma in the left external ear canal. Additionally, a suspicious lymph node in the parotid gland region was noted and excised during revision ear canal surgery. Histopathological evaluation of the excised tissue confirmed now the presence of an invasive SCC. These findings suggested malignant transformation of the inverted papilloma within the left ear canal, with concurrent lymph node metastasis (see **Figures 3D–F**).

In December 2017, the patient underwent revision sinus surgery and revision ear canal surgery. Histopathological analysis confirmed the presence of SCC in both the ear canal and the sinonasal cavities.

Following the revision surgery, the patient was treated with adjuvant CRT from January to March 2018, comprising a total dose of 63 Gy with concurrent cisplatin. The treatment targeted the left nasal cavity, left maxillary sinus, left external ear canal, and ipsilateral cervical lymph nodes.

In April 2019, the patient was presented with persistent chronic pain in the left mastoid region, a newly identified suspicious mass in the left external ear canal and mastoid area, and new-onset facial paralysis. These findings prompted the decision to proceed with revision surgery with histopathological examination revealing a recurrence of a poorly differentiated, non-keratinizing SCC.

The detection of recurrent disease along with a newly identified cerebral lesion in the left temporal lobe on MRI (see **Figure 3C**)—highly suggestive of metastasis with associated midline shift—prompted the decision to pursue a combined otolaryngological and neurosurgical intervention. In November 2019, the patient underwent a left-sided parotidectomy with hypoglossal nerve jump anastomosis and resection of the temporal metastasis. The postoperative course was complicated by the development of a cavernous sinus thrombosis, necessitating prolonged intensive care management.

In January 2020, the patient succumbed to the disease. Postmortem analysis of FFPE tissue samples obtained during surgery in April 2019 was reinterpreted as being consistent with *DEK::AFF2* carcinoma which was then confirmed by detection of the DEK-AFF2 fusion, underscoring its role in the aggressive disease progression.

## Discussion

The presented cases underscore the formidable clinical challenges posed by *DEK::AFF2* fusion-associated sinonasal carcinomas, characterized by aggressive local invasion and a high propensity for recurrence. In both cases, diagnostic confirmation of DEK::AFF2 fusion was significantly delayed, primarily due to initial misclassification as inverted papilloma, a pitfall commonly encountered given the overlapping morphological features. Histopathological evaluation revealed that *DEK::AFF2* carcinomas frequently exhibit a complex architecture, including endophytic and exophytic, often papilloma-like growth patterns. The neoplastic cells display transitional epithelium with eosinophilic to amphophilic cytoplasm, absent or minimal keratinization, sporadic compact keratin pearls, monotonous nuclei, and a conspicuous infiltration of neutrophils or stromal lymphocytes (3, 5, 9). Consistent with previously reported findings, both cases presented in this report exhibited a combination of exophytic and endophytic architectural patterns, along with focal hyperparakeratotic squamous metaplasia.

However, the deceptively bland histological features raise critical challenges in distinguishing *DEK::AFF2* fusion-associated sinonasal carcinomas from inverted sinonasal papillomas based solely on morphology. Immunohistochemical analysis and genetic profiling, particularly evaluating the mutational status of *EGFR* and *KRAS*, may offer critical diagnostic insights (3). Although these mutations are characteristic of sinonasal papillomas, they have been consistently absent in reported cases of *DEK::AFF2* fusion, underscoring their potential utility in differential diagnosis (1, 3, 10). In this context, the absence of EGFR and KRAS mutations may serve as a critical molecular indicator warranting further investigation for gene fusion events, such as DEK::AFF2, particularly in clinically aggressive lesions that exhibit deceptively indolent histologic features. Given the diagnostic challenges associated with DEK::AFF2 sinonasal carcinomas, alternative detection strategies beyond the widely used RNA sequencing are being explored to enhance diagnostic sensitivity and specificity. Notably, Kuo et al. evaluated an immunohistochemical assay targeting the AFF2 C-terminus, demonstrating 100% sensitivity and specificity for DEK::AFF2 sinonasal carcinomas. These findings suggest that AFF2 immunohistochemistry represents a highly sensitive, specific, and cost-effective ancillary tool that may aid in the diagnostic workup of DEK::AFF2 sinonasal carcinomas (11).

Nonetheless, the decision to pursue fusion testing—irrespective of the methodology—should be guided by a multidisciplinary framework, integrating histopathological uncertainty, patterns of clinical progression, and radiographic evidence of aggressive behavior. Implementing such a diagnostic algorithm may facilitate earlier detection of DEK::AFF2 fusion-associated carcinomas and inform timely, individualized therapeutic strategies.

Given the novelty of *DEK::AFF2* fusion-associated carcinomas, standardized treatment protocols are currently lacking. The optimal therapeutic approach—whether surgical intervention, systemic therapy, radiation, or a combination thereof—remains undefined. Consequently, case reports such as this one are invaluable for providing insights into emerging therapeutic strategies and raising awareness among clinicians about this rare malignancy.

In this report, we demonstrate that neoadjuvant treatment with gemcitabine and cisplatin can yield an exceptional response, as evidenced in our first case. To our knowledge, this represents the first documented complete radiographic remission in response to neoadjuvant chemotherapy in a DEK::AFF2 fusion-associated carcinoma. This finding contrasts with the outcomes reported by Yang et al. who reported a case of *DEK::AFF2* fusion carcinoma with lung metastases demonstrating an exceptional response to PD-1 inhibition therapy (pembrolizumab), despite negative PD-L1 expression. Notably, this response occurred following disease progression under prior treatment with platinum-based chemotherapy and 5-fluorouracil, which failed to achieve disease control after one year (7). The molecular basis for the exceptional response to immunotherapy may be attributed to gene fusion-associated neoantigens, which enhance tumor-specific T cell activation (7).

Beyond the adjuvant, radiotherapeutic, or combined chemoradiotherapeutic strategies outlined by Kou et al. (11) and the immunotherapeutic approach described by Yang et al. (7), there are currently no standardized or widely accepted treatment algorithms for DEK::AFF2 fusion-positive carcinomas. No alternative therapeutic modalities—other than the neoadjuvant treatment employed in this case—have been documented in the current literature. This highlights the urgent need for collaborative multicenter registries, further molecular characterization, and prospective studies to refine diagnostic criteria and develop evidence-based therapeutic frameworks for this aggressive tumor entity.

## Data Availability

The original contributions presented in the study are included in the article/**Supplementary Material**. Further inquiries can be directed to the corresponding author.
