# Supplementary material for: Case Report: “DEK::AFF2 fusion associated sinonasal carcinomas: a novel oncogenic driver and emerging therapeutic strategies”
Source: Front Immunol. 2025 Jul 4;16:1611790. doi: 10.3389/fimmu.2025.1611790 (PMC12271193; doi:10.3389/fimmu.2025.1611790)
Supplement: Supplementary file 1 [file DataSheet1.docx]

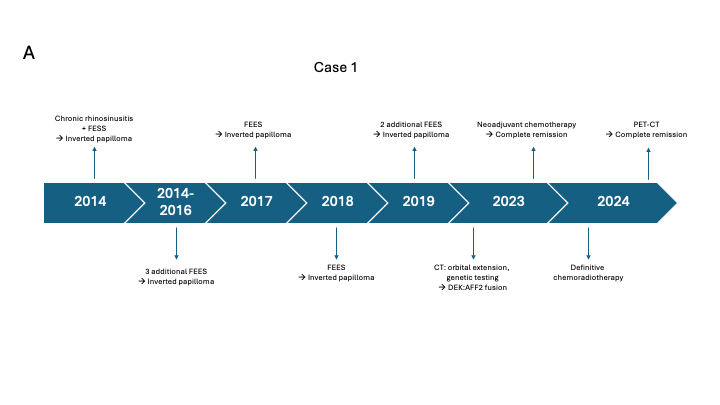


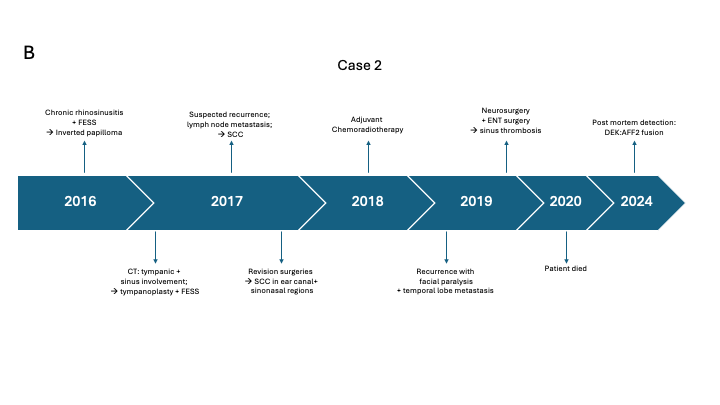


**Supplementary Figure 1.**Timeline illustrating key clinical events and therapeutic interventions for (A) Patient 1 and (B) Patient 2.
